# Efficient targeted tumor imaging and secreted endostatin gene delivery by anti-CD105 immunoliposomes

**DOI:** 10.1186/s13046-018-0712-8

**Published:** 2018-03-02

**Authors:** Huiqin Zhuo, Baoshi Zheng, Jianming Liu, Yong Huang, Huiling Wang, Duo Zheng, Naiquan Mao, Jinyu Meng, Sufang Zhou, Liping Zhong, Yongxiang Zhao

**Affiliations:** 1 Department of Gastrointestinal Surgery, Institute of Gastrointestinal Oncology, Zhongshan Hospital, Xiamen University, Xiamen, Fujian 361004 China; 2 National Center for International Research of Biological Targeting Diagnosis and Therapy/Guangxi Key Laboratory of Biological Targeting Diagnosis and Therapy Research/Collaborative Innovation Center for Targeting Tumor Diagnosis and Therapy, Guangxi Medical University, Nanning, Guangxi 530021 China; 3 Department of Cardiothoracic Surgery, First Affiliated Hospital, Guangxi Medical University, Nanning, Guangxi 530021 China; 4 The Third Xiangya Hospital, Central South University, Changsha, 410083 China; 5 Department of Basic Medicine, Shenzhen Key Laboratory of Translational Medicine of Tumor, School of Medicine, Shenzhen University, Shenzhen, Guangdong 518000 China; 6 Biomedical Polymers Laboratory, Soochow University, Suzhou, Jiangsu 215123 China

**Keywords:** Anti-CD105 immunoliposome, Tumor vascular targeting, In vivo imaging, Secreted endostatin, Gene therapy

## Abstract

**Background:**

Anti-CD105 mAb-conjugated immunoliposomes, loaded with secreted mouse endostatin gene, were developed for targeted tumor imaging and antiangiogenic gene therapy.

**Methods:**

The liposomes were investigated for size, zeta-potential, lipid content, antibody binding ability, and pcDNA loading capacity. The ability of immunoliposomes to target tumor-derived endothelial cells and perform gene transfer in vitro was measured and their basic biocompatibility was evaluated. A nude mouse/breast cancer xenograft model was used to examine the tumor internalization of fluorescent-labeled liposomes and the clinical potential of immnuoliposomes loaded with pcDNA3.1-CSF1-endostatin.

**Results:**

Loaded immunoliposomes were homogenously distributed with a well-defined spherical shape and bilayer, diameter of 122 ± 11 nm, and zeta potential + 1.40 mV. No significant differences were observed in body weight, liver index, oxidative stress, or liver and kidney function in mice after liposomes exposure. The addition of CD105 mAb to liposomes conferred the ability to target tumor-derived endothelial cells in vitro and in vivo. Systemic intravenous administration of fluorescent immunoliposomes in the xenograft model resulted in selective and efficient internalization in tumor vasculature. Treatment of mice with pcDNA3.1-CSF1-endostatin-loaded immunoliposomes suppressed tumor growth by 71%.

**Conclusions:**

These data demonstrate the advantages of using anti-CD105 mAb-conjugated immunoliposomes to enhance tumor targeting, imaging, and gene transfer applications.

## Background

Given the vital importance of angiogenesis in tumor initiation, development, and metastasis, significant effort has gone into angiogenesis-related research over the past decade, including molecular imaging techniques and targeted drug/gene therapy. Endoglin (CD105, 180 kDa), a disulfide-linked homodimeric cell membrane glycoprotein, is critical for appropriate blood vessel development [1–3]. Tumor-associated endothelial cells (TECs) have been reported to proliferate 20–2000 times more rapidly than those from normal tissues do, indicating that CD105 may represent a useful prognostic and diagnostic marker or therapeutic target [4, 5]. Recently, a series of reports on CD105 imaging described labeling with anti-CD105 antibodies for visualizing CD105 expression during in vivo targeted imaging of solid malignancies [6–10]. Radiolabeled monoclonal anti-CD105 antibodies or scFv-binding have been reported to significantly enhance tumor uptake and permit efficient tumor visualization in xenograft animal models by using positron emission tomography (PET), single photon emission computed tomography/computed tomography (SPECT/CT) [6–8], or PET/near-infrared fluorescence (NIRF) imaging [9, 10]. Increasing number of studies have reported that targeting of CD105 on tumor-associated blood vessels may represent an efficient strategy for in vivo imaging of solid malignancies, regardless of their histological origin.

Liposome (Lp) is versatile carrier systems for the delivery of cytotoxic drugs or genes, and provides a platform for enhancing the efficiency of delivery and targeting of therapies to tumor tissues [11–16] PEGylated Lp, in which polyethylene glycol lipids are incorporated into the lipid bilayer, have the advantage of preventing loaded gene degradation and clearance [12]. Furthermore, when combined with tumor-specific markers, the efficiency of targeted delivery can be significantly enhanced [11, 13]. Nanographenes synthesized to target CD105 with anti-CD105 mAb or microbubbles conjugated with anti-vascular endothelial growth factor (VEGF) mAb have been reported to be specifically directed to tumor microvasculature in vivo [15, 16]. The significantly improved tumor targeting efficiency of a transferrin-conjugated Lp system described in our previous work [13] may be utilized for tumor neovasculature-targeted imaging and/or therapeutic gene delivery in vivo. To the best of our knowledge, little has been reported to date about targeted CD105 Lp delivery systems incorporating both anticancer genes and fluorescent dyes in different compartments of a single vesicle.

The aim of this study was to establish a novel, dual-function, targeted CD105 immunoliposome (ILp) possessing the capacity for targeted tumor imaging and antiangiogenesis gene-specific delivery in animal tumor models. To evaluate the biodistribution and tumor-targeting efficacy of anti-CD105 ILp in tumor-bearing mice, serial imaging of Lp labeled with X-SIGHT 670 Large Stokes Shift Dye (LSS670) was performed using an in vivo imaging system. The endostatin gene was selected for use in our study because of its established antiangiogenic properties [17] and potential to overcome the issue of protein instability. The inhibitory effect of ILp loaded with the mouse endostatin gene (mES) was examined in MDA-MB-231 xenograft tumor models. To validate the in vivo data, various in vitro and ex vivo studies were also carried out to confirm the targeted imaging and gene delivery properties of ILp.

## Methods

### Materials

POPC, DDAB (dimethyldioctadecyl ammonium bromide salt), DSPE-PEG_2000_ (1,2-distearoyl-sn-glycero-3-phosphoethanolamine-N-[amino (polyethylene glycol)-2000] ammonium salt), and DSPE-PEG_2000_-maleimide (1,2-distearoyl-*sn*-glycero-3- phosphoethanolamine-N-[maleimide (polyethylene glycol)-2000] ammonium salt) were purchased from Avanti-Polar Lipids Inc. (Alabaster, AL, USA). Purified anti-mouse CD105 (clone: MJ7/18) and isotype-matched control rat IgG monoclonal antibodies were purchased from eBioscience (San Diego, CA, USA). The fluorescent molecule, LSS670, was purchased from Kodak (Carestream Health, Inc., Rochester, NY, USA).

### Plasmid preparation

The upstream and downstream primers for mEndo were 5′-CCCAAGCTTGCCACCATGCATACTCATCAGGACTTTCAG-3′ and 5′-CCGGAATTCCTATTTGGAGAAAGAGGTCATGAAG-3′, respectively. A nucleotide sequence encoding the colony-stimulating factor 1 (CSF1) signaling peptide was synthesized by Invitrogen Biotech Co. (Guangzhou, China). The DNA segment and pcDNA3.1+ vector were digested and ligated to create the recombinant plasmid, pcDNA3.1-ES. Next, the CSF1 signal peptide sequence was added to the N-terminal of ES to create pcDNA3.1-CSF1-mES. The upstream and downstream primers for CSF1 were 5′-CCCAAGCTTGCCACCATGACCGCGCCGGGCGCCGCCGGGCGCTGCCCTCCCACGACATGGCTGGGCTCCCTGC-3′ and 5′-CCGGAATTCGGTGATACTCCTGCTCGCCAGGAG ACAGACCAACAACAGCAGGGAGCCCAGCCATG-3′, respectively. EGFP was encoded in pcDNA3.1+ as a reporter gene, and used to evaluate the efficiency of gene delivery to TECs. Plasmid DNA was prepared using an EndoFree Giga kit (Qiagen, Hilden, Germany), according to the manufacturer’s instructions. The concentration of plasmid DNA was measured using an Infinite M1000 Pro microplate reader (Tecan, Durham, NC, USA).

### Cell culture

An MDA-MB-231 cell line stably expressing luciferase (MDA-MB-231-Luc) was established in our lab [13]. Cell lines MDA-MB-231-Luc, HeLa, LTEP-α-2, and HEK293T were all cultured in Dulbecco’s modified Eagle’s medium (DMEM), fetal bovine serum (FBS), penicillin (100 U/mL), and streptomycin (100 μg/mL). TECs were derived from Lewis lung carcinoma tumors in C57BL/6 mice and isolated via magnetic-activated cell sorting (MACS) of anti-CD105 Ab coupled to magnetic beads, according to the method of Zhuo et al. [18, 19]. TECs were grown in M131 medium supplemented with microvascular growth supplement (MVGS) (Cascade Biologics, Portland, OR, USA).

### Animals

Kunming mice, BALB/c nude mice, and NOD/SCID mice (female, 10-week-old) were purchased from the Model Animal Research Center of the Medical College of Xiamen University and housed under laminar flow and sterile conditions. Animal care and handling were performed in accordance with the guidelines for the Care and Use of Laboratory animals, and the animal study protocol was approved by the Institutional Animal Care and Use Committee of Xiamen University.

### Preparation of PEGylated anti-CD105 ILp

Lp was prepared as described previously, with some modifications [13]. Briefly, chloroform solutions of POPC, DSPE-PEG_2000_-maleimide, DSPE-PEG_2000_, and DDAB (at a molar ratio of 93:1:3:3) were mixed in a glass tube. A thin uniform lipid film was created by evaporation under a constant nitrogen flow. Dried lipids were rehydrated with 0.2 mL of PBS buffer and sonicated in a water bath for 5 min. An aliquot of plasmid DNA (400 μg) or calcein solution (2 μL, 0.01 mol/L) was added to the lipid solution. Ethanol and 8 mM calcium solution were slowly added, with final concentrations of 35% ethanol and 4 mM Ca^2+^, respectively. The vials were subjected to 5 freeze-thaw cycles. Lp was extruded with a hand-held extruder (Avestin, Ottawa, Canada) through two polycarbonate membranes (100-nm filters for 20 times and 50-nm filters for 21 times). The Lp was dialyzed with Slid-A-Lyzer dialysis cassettes (10,000 molecular weight cutoff [MWCO]) (Pierce, Rockford, IL, USA) against HEPES buffer (25 mM HEPES, 140 mM NaCl, pH 7.0) for 2 h, with buffer replenished after 1 h.

Antibody coupling was conducted at a protein:lipid molar ratio of 1:1000. The anti-CD105 antibody was chemically activated by reaction with 2-iminothiolane (200 μL, 7 mmol/L, Sigma Chemical Co. St. Louis, MO, USA) in sodium borate buffer (50 mM, pH 9.4) in the dark for 2 h at room temperature. After washing twice with HEPES buffer by ultrafilter-centrifugation (Microcon Ultracel YM-30, 30 kDa MWCO; Millipore, Bedford, MA, USA), the antibody was incubated overnight with Lp. The vesicles were subjected to size-exclusion chromatography with a Sepharose CL-4B column (GE Healthcare, Saclay, France), and eluted with PBS solution. The Lp fractions were collected, filtered through a 0.22-μm polyvinylidene fluoride (PVDF) membrane filter (Millipore), and stored at 4 °C until use. All Lp eluate fractions were collected and freeze-dried for the detection of uncoupling antibodies using a Bradford assay kit. The DNA encapsulation efficiency of Lp was measured as reported previously [13]. All types of Lp were stable in suspension for up to 3 months at 4 °C.

### Particle characterization

Particle sizes were measured three times in 150 mmol/L NaCl using DLS at a 173° angle on a Zetasizer Nano-Zs (Malvern Instruments, London, UK) using the Contin algorithm at 25 °C. Zeta potentials were measured in 150 mmol/L NaCl (pH 7.0) with a laser electrophoresis zeta potential analyzer LEZA-700 (Otsuka Electronics, Osaka, Japan). DNase I (Sigma) digestion was performed to remove the exterior plasmid according to the method of Oliveira et al. [20].

TEM was used to analyze the morphology of the Lp. A drop of the Lp sample was placed on a 100-mesh copper grid and excess fluid was removed. A 2% phosphotungstic acid solution (pH 7.4) was dropped onto the grid and dried for 12 h in a desiccator. The sample was subsequently observed by TEM (JEM2100HC, JEOL Ltd., Tokyo, Japan).

### Assessment of stability and DNA protection of ILp/pcDNA

Two experiments were performed for the assessment. 1) ILp/pcDNA was incubated with DMEM, DMEM supplied with 10% FBS, or human serum for 2 to 14 h at 37 °C. The plasmid DNA leakage ratio was calculated according to our previous report [13]. 2) DNase I digestion was performed according to the method of Oliveira et al. [20]. Briefly, 1 μg plasmid (control) and ILp/pcDNA (corresponded to 1 μg DNA) was submitted to DNase I action (2 U DNase I per μg of DNA) for 10 min, 0.5 h or 1.0 h at 37 °C, followed by incubation with 0.5 M EDTA to stop the enzyme activation. 5% Triton X-100 was used to release DNA from the ILp/pcDNA after 1 h DNase digestion. The samples were analyzed by 1% agarose gel electrophoresis.

### Quantitative analysis of POPC using UHPLC-MS-MS

An ultra-high performance liquid chromatography tandem mass spectrometry (UHPLC-MS-MS, Nexera UHPLC System, Shimadzu, Kyoto, Japan) method has been developed for rapid quantitative analysis of POPC in Lp. POPC has a maximum absorption peak at 205 nm, with *m/z* 761. A stock solution of POPC was prepared in chloroform, to a final concentration of 8 mg/mL. POPC of Lp was dried completely in a desiccator and extracted using 100% methanol. Chromatographic separations were carried out using a Shimadzu LCMS-8050 triple quadrupole mass spectrometer equipped with a Shimadzu Nexera X2 UHPLC system. POPC was separated on a Shim-pack XR-ODSIII (2.0 mm i.d. × 75 mm, 1.6 μm) column, monitored with SPD-M20A r at 205 nm. Methanol (100%) was used as the eluting solution at a flow rate of 0.2 mL/min. The total run time was 25 min. The column oven temperature was 40 °C, and the injection volume was 5 μL. Positive ion electrospray mass spectrometry was used for the measurement of POPC with the following parameter settings: nebulizer flow rate, 2 L/min; dryer flow rate, 10 L/min; DL temperature, 250 °C; heating block temperature, 400 °C; and ion mode, ESI.

### Cellular uptake study

Cellular uptake of complexes was determined in CD105 positive cells (TECs) using calcein-loaded ILp. The cells were treated with calcein complexed Lp for 4 h at 37 °C in complete medium. After incubation, Lp was removed and the cells were washed four times and fixed with 4% formaldehyde for 30 min. The cell nuclei was counterstained with 4,6-diamidino-2-phenylindole (DAPI; Invitrogen, Karlsruhe, Germany). The cells were visualized under confocal microscopy (Zeiss LSM 780, Carl Zeiss, Jena, Germany). Cells cultured in a 6-well plate were treated with calcein-loaded Lp or ILp with isotype mAb cell pretreatment for 1 h, containing 100 μg lipid diluted in 1 mL of medium for 2 h at 37 °C. Transfection efficiency was determined using a Gallios flow cytometer (Beckman Coulter Inc., Brea, CA, USA). Total of 10,000 events based on the front scatter (FSS) and side scatter (SSC) gate were analyzed and displayed by colored histograms.

### In vitro gene transfection

The cells were incubated with a medium containing naked pcDNA3.1-EGFP, Lp/pcDNA3.1-EGFP, or ILp/pcDNA3.1-EGFP complex under standard incubation conditions for 5 h. The medium was then replaced, and the cells were cultured for further 48 h. Cells harboring and expressing integrant were viewed by fluorescence microscopy based on EGFP and analysis by flow cytometry.

Expression of secreted mES was detected in HeLa, LTEP-α-2, and HEK293T cells using a mouse endostatin ELISA commercial kit (LifeSpan BioSciences, Seattle, WA, USA) according to the manufacturer’s instructions. The cells were transfected with 4 μg/plate of pcDNA3.1-CSF1-mES using Attractene transfection reagent (Qiagen), and the culture medium was collected at 24, 48, and 72 h.

### Evaluation of in vivo toxicity

Forty-eight Kunming mice (22–25 g, 5–6 weeks old) were randomly allocated to four groups with 12 mice in each group: PBS, Lp, Lp/pcDNA, and ILp/pcDNA (with a POPC concentration of 10 mg/kg). Every four days, for a total of four doses of 200 μL solution for each mouse, the appropriate treatment was injected into the tail vein. Behavior and any abnormal symptoms were monitored daily. Six mice in each group were sacrificed at 5 and 17 days after injection. Anticoagulated blood samples (with the addition of heparin) were collected from the vena ophthalmica and centrifuged at 3000 rpm for 15 min. The resulting plasma was collected and stored at − 80 °C until use. The liver index (liver weight/body weight [g/g]) was calculated, and a section of liver tissue was stripped and immediately fixed in 4% formaldehyde for hematoxylin-eosin staining. The remaining liver tissue was weighed and homogenized in ice-cold buffer to yield 10% (*w*/*v*) homogenate for reduced glutathione (GSH) concentration assay and total superoxide dismutase (SOD) activity assays. The concentration of GSH and total SOD activity were determined using detection kits purchased from the Nanjing Jiancheng Bioengineering Institute (Nanjing, Jiangsu, China).

The biochemical indices, ALT, AST, BIL, CRE and URE were measured using the Roche Cobas 8000 automatic biochemical analyzer (Roche Diagnostics, Basel, Switzerland). TNF-α levels were determined using a commercial ELISA kit (eBioscience, San Diego, CA, USA) according to the manufacturer’s instructions.

### In vivo optical imaging

As a tumor model, the MDA-MB-231-Luc breast cancer cell line was used, which expressed the luciferase gene (Luc). With the cell line, sites and sizes of xenografts were visualized by bioluminescent imaging in vivo, and the merging images of bioluminescence and fluorescence were used to estimate the tumor-targeting ability of liposomes. Tumors were established by subcutaneously injecting 5 × 10^6^ MDA-MB-231-Luc cells under the front right axilla of immunocompromised 10-week-old female Balb/c nude mice. The tumor size was monitored every other day, and the mice were used for in vivo experiments when the tumor diameter reached 0.8–1.0 cm (typically 2 weeks after inoculation). Xenograft-bearing mice were randomly divided into two groups (*n* = 3 per group) and injected with LSS670-labeled Lp or LSS670-labeled ILp. The labeling reactions were carried out according to the manufacturer’s protocol. The animals were anesthetized using 3.5% isoflurane and maintained unconscious using 2.0–2.5% isoflurane during injection and imaging.

Optical imaging was performed with the Kodak in vivo FXPro imaging system (Carestream Health Inc., New York, USA), which combines multispectral fluorescence, luminescence, and digital X-ray capabilities in a single system. The imaging system consisted of a dark chamber with gas anesthesia inlet and outlet ports. The excitation and emission filters were set at 650 and 700 nm, respectively. All images were acquired using the following parameters, which were optimized to improve the signal-to-noise ratio: 60,000 s exposure time; 2 × 2 binning; 60 s acquisition time; 120 × 120 mm field of view; and f 2.25 aperture stop.

The animals were carefully wiped with alcohol to remove any fluorescing contaminants, and baseline imaging was performed. Nanoparticles (100 μL) were injected through the tail vein and in vivo fluorescence scanning was performed at various time points after injection. For bioluminescent imaging, the light-sensitive substrate D-luciferin was administered by intraperitoneal injection of approximately 2.5 mg luciferin/kg body weight. The exposure time was 3 min, with 4 × 4 binning.

Four additional tumor-bearing mice were intravenously injected with LSS670-labeled Lp or LSS670-labeled ILp and euthanized at 12 h post injection (at peak tumor uptake, based on imaging results). Biodistribution studies were carried out to validate quantitative fluorescent dye uptake values derived from ex vivo fluorescent scans. Tumor and major organs/tissues were collected, and fluorescence was measured and analyzed by the imaging system. Tumor xenografts were also frozen for confocal microscopy analysis.

### In vivo targeted therapy with pcDNA3.1-CSF1-mES-loaded ILp

To establish subcutaneous (*s.c.*) tumors, 5 × 10^6^ MDA-MB-231 cells in 0.2 mL of PBS were injected into the mammary fat pad of NOD/SCID mice. The mice were randomly allocated to four groups (*n* = 7), intravenously (*i.v.*)-treated with 150 μL of PBS, naked pcDNA3.1-CSF1-mES, anti-CD105 mAb (105 μg, an amount equal to that in ILp), or ILp/pcDNA3.1-CSF1-mES. The quantity of pcDNA3.1-CSF1-mES corresponded to 50 μg DNA. The animals were treated four times, on days 1, 5, 9, and 13 after tumor establishment. Before gene therapy, MDA-MB-231 tumor-bearing mice were treated with pcDNA3.1-EGFP-loaded ILp in order to evaluate the ILp in vivo transfection capacity. Then, the tumor tissues were stripped for the detection of EGFP expression using confocal microscopy of frozen slices.

Tumors were measured with Vernier calipers at 3-day intervals after the inoculation of tumor cells. Tumor volume was calculated according to the formula d1 × (d2)^2^ × 0.5, where d1 is the largest diameter and d2 is the perpendicular diameter. The mice were monitored daily for survival.

### Statistical analysis

SPSS Viewer software v20.0 (SPSS, IL, USA) was used for variance analysis. A two-tailed *P* < 0.05 was considered statistically significant. Data were represented as the mean ± SD.

## Results

### Characterization of liposomes

**Fig. 1:**
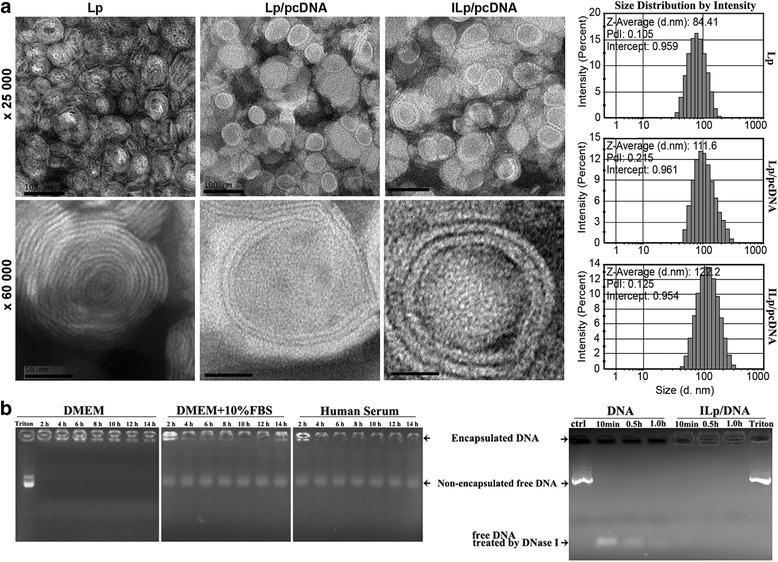
Particle characteristics, stability and DNA protection property. **a** Transmission electron microscopy images and dynamic light scattering particle size distribution for Lp, pcDNA3.1-CSF1-mES loaded Lp (Lp/pcDNA), and pcDNA3.1-CSF1-mES loaded immunoliposomes (ILp/pcDNA). Scale bar: 100 nm (× 25,000), and 50 nm (× 60,000). **b** Stability and DNA protection property of ILp/pcDNA. ILp/pcDNA was incubated with DMEM, DMEM supplied with 10% FBS medium, or human serum for 2, 4, 6, 8, 10, 12 and 14 h at 37 °C. 1 μg plasmid (control) and ILp/pcDNA (corresponded to 1 μg DNA) was submitted to DNase I action (2 U DNase I per μg of DNA) for 10 min, 0.5 h or 1.0 h at 37 °C, followed by incubation with 0.5 M EDTA to stop the enzyme activation. The samples were analyzed by 1% agarose gel electrophoresis. Degraded DNA produced by DNase I digestion migrated in the bottom of gel. Encapsulated DNA remained in the well in concordance with ILp not being able to migrate through the agarose matrix. 5% Triton X-100 was used to release DNA from the ILp/pcDNA after 1 h DNase digestion (last lane)

PEGylated Lp were homogenously distributed as individual nanoparticles with a well-defined spherical shape and diameter of 84 ± 14 nm (Fig. 1). When plasmid DNA was incorporated into PEGylated Lp, the average particle size was 112 ± 12 nm. Anti-CD105 mAb binding increased the Lp size to about 122 ± 11 nm, which corresponds to the dynamic light scattering (DLS) measurements. As shown by transmission electron microscopy (TEM), Lp (multilayers) not only has hydrophobic dissections but also hydrophilic dissections with a small lumen, so in theory two components can incorporate together that highly improved the carrier efficiency. As expected, the lipid membrane structure changed to a bilayer with a notable larger lumen following pcDNA loading (Fig. 1a). There was no significant difference in size and structure before and after antibody binding. Polydispersity index (PDI) of Lp, Lp/pcDNA and ILp/pcDNA was 0.105, 0.215 and 0.125, respectively. All values were within the recommended size for medical applications, which is a PDI of less than 0.3 [21].

PEGylated Lp had a positive zeta potential of + 4.4 mV. The value of Lp/pcDNA was + 5.98 mV. The Lp/pcDNA had been dialyzed against HEPES buffer, and digested with DNase I to remove the plasmid on liposome surface before detection. This value became + 1.40 mV after anti-CD105 antibody conjugation. The zeta potential of ILp/pcDNA with or without DNase I digestion, was verified to be almost equal to each other, indicated the exterior plasmid had been completely removed from the liposome surface, after the procedure of size-exclusion chromatography. The encapsulation efficiency of ILp-entrapped pcDNA3.1-CSF1-mES was 72.5 ± 3.5%.

The stability of ILp/pcDNA was assessed in the human serum and DMEM with or without FBS for up to 14 h. As shown in Fig. 1b, ILp/pcDNA was quite stable in DMEM medium, but with some leakage in the presence of FBS. The leaked plasmid DNA was extracted, and the leakage ratio (%) was precisely calculated from samples in parallel. The ratio was approximately 11% after incubated with DMEM in the presence of 10% FBS for 2 h, and the increasing value was detected in the following 12 h but far more slowly than before. The leakage ratio was close to 18% after 14 h of incubation. Similar results were observed in the presences of human serum. DNA protection was estimated by incubation free plasmid DNA or ILp/pcDNA with DNase I. The results presented in Fig. 1b. showed that control DNA (free plasmid) was completely digested and degraded DNA produced by DNase I digestion migrated in the bottom of gel. But encapsulated DNA remained in the well in concordance with ILp not being able to migrate through the agarose matrix, without oligomer fragment migration in the bottom of gel. Triton X-100 was used to release DNA from the ILp/pcDNA after 1 h DNase digestion (last lane). The ILp offered the encapsulated DNA more resistance to DNase I digestion.

**Fig. 2:**
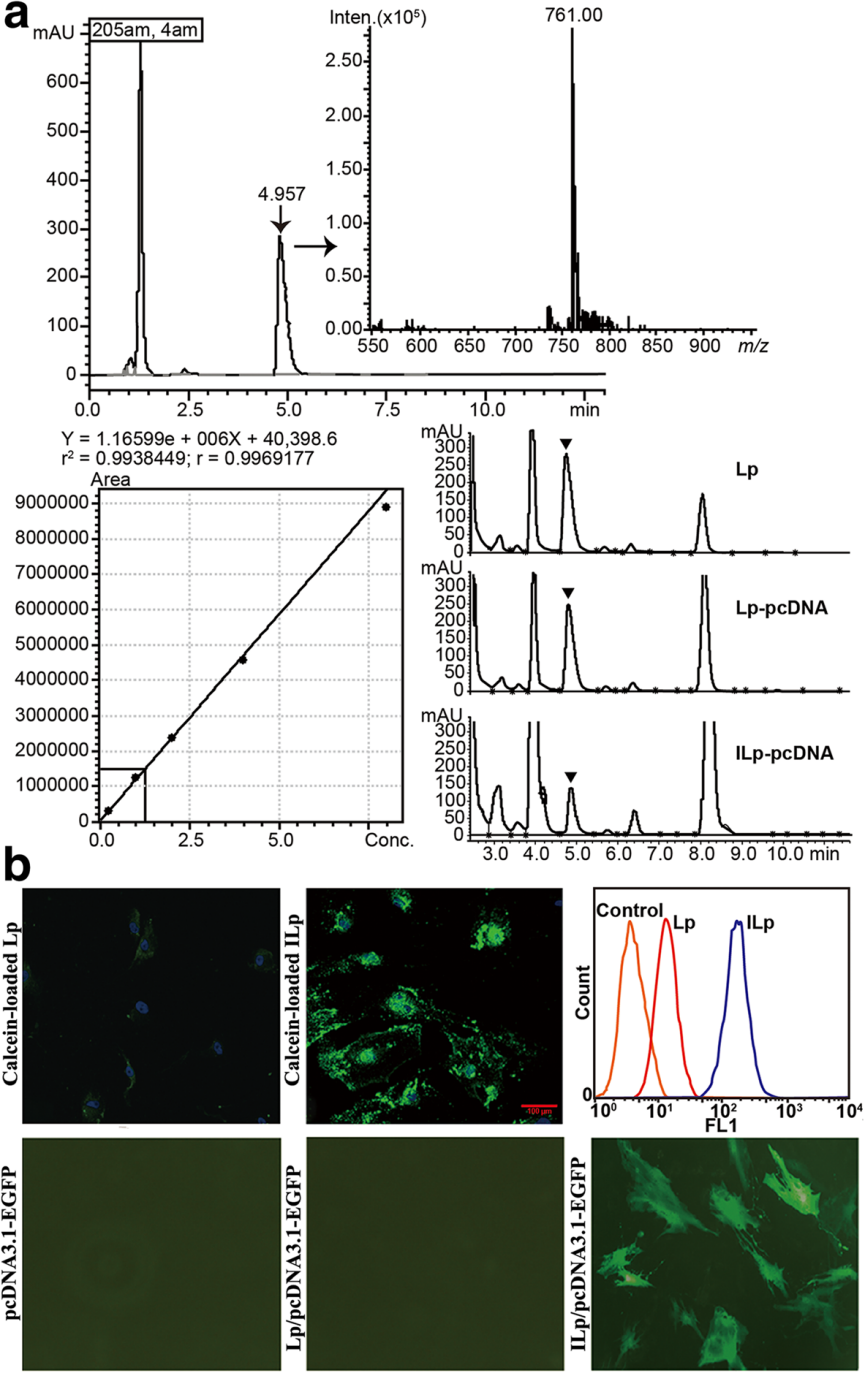
Characterization of liposomes. **a** Rapid screening and quantitative analysis of 1-palmitoyl, 2-oleoyl phosphatidylcholine (POPC) in Lp by UHPLC-MS-MS. POPC had a maximum absorption peak at 205 nm, with *m/z* 761. A stock solution of POPC was prepared in chloroform, to a final concentration of 8 mg/mL. Working standards were prepared by serial dilution of stock solutions. Lp POPC was dried in a desiccator and extracted with 100% methanol. Chromatographic separations were carried out using a Shimadzu LCMS-8050 triple quadrupole mass spectrometer equipped with a Shimadzu Nexera X2 UHPLC system. In vitro cellular association and in vivo toxicity of Lp with or without pcDNA loading. **b** Confocal fluorescence images showing uptake of calcein-loaded Lp or ILp by primary tumor-derived microvascular endothelial cells (TECs; DAPI/nuclei, blue; calcein, green). Flow cytometry histograms of cellular fluorescence uptake. Negative control binding experiments were performed using isotype-matched controls or undecorated Lp. Total of 10,000 events based on the front scatter (FSS) and side scatter (SSC) gate were analyzed and displayed by colored histograms. Orange line, control; red line, Lp; blue line, ILp. Fluorescence micrographs of TECs transfected with pcDNA3.1-EGFP, Lp/pcDNA3.1-EGFP, and ILp/pcDNA3.1-EGFP

Since 1-palmitoyl, 2-oleoyl phosphatidylcholine (POPC) is the most important constituent of Lp, an UHPLC-MS-MS method has been developed for the rapid quantitative analysis of POPC, according to its maximum absorption peak at 205 nm with *m/z* 761 (Fig. 2a). The retention time for POPC was 4.957 min, and the standard curve was linear (r^2^ = 0.994). POPC in Lp, Lp/pcDNA, and ILp/pcDNA was detected and estimated according to the standard curve. The recoveries were 99.32, 97.45, and 71.10%, and the concentrations were 28.05, 12.46, and 7.42 mg/mL, respectively. On comparing recovery, almost all POPC was used for Lp preparation. However, dialysis to remove unenveloped pcDNA caused a small amount of loss and nearly one-third of POPC was lost following size-exclusion chromatography. Here, every milligram of POPC contained 20 μg pcDNA and 42 μg anti-CD105 mAb in the prepared ILp/pcDNA.

### In vitro cellular association of ILp

The fluorescent signal on TECs was minimal for calcein-loaded Lp, indicating low nonspecific binding in the cell culture (Fig. 2b). The ILp was recognized and internalized by the TECs, and partly located near the nucleus in a granular pattern, indicating active transport into the cell. On the basis of fluorescence-activated cell sorting (FACS) analysis of CD105 positive cells, isotype-matched controls or undecorated Lp was used for negative control binding experiments. These negative controls resulted in significantly lower internalization compared with ILp, confirming the confocal microscopy observations. Furthermore, results indicated that conjugation to Lp did not alter the antigen-binding affinity or specificity of anti-CD105 mAb.

Cells expressing EGFP were directly visualized and imaged using fluorescence microscopy (Fig. 2b) and further evaluated by flow cytometry at 48 h after incubation. EGFP expression was minimal when the cells were transfected with naked plasmid DNA (negative control) or EGFP-containing Lp, which suggested that TEC did not easily internalize naked EGFP or EGFP carried by Lp, even when Attractene transfection reagent was used (data not shown). However, the transfection efficiency of ILp/pcDNA3.1-EGFP increased by 35%, which was notably better than that of nontargeted Lp.

The expression of secreted mouse endostatin (mES) was detected in culture medium of HeLa, LTEP-α-2, and HEK293T cells by using ELISA instead of TECs. Because of the TEC cell death induced by the gradual expression of endostatin several hours after transfection, for its established antiangiogenic properties. mES in the medium of HeLa cells transfected with pcDNA3.1 was below the detection limit, but a total of 96.22, 148.32, and 151.57 ng of secreted mES was detected at 24, 48, and 72 h after transfection with pcDNA3.1-CSF1-mES, respectively. Almost all mES was secreted at 48 h after transfection, and similar amounts were detected in LTEP-α-2 and HEK293T cells, 121.79 and 166.49 ng, respectively.

### In vivo toxicity

**Fig. 3:**
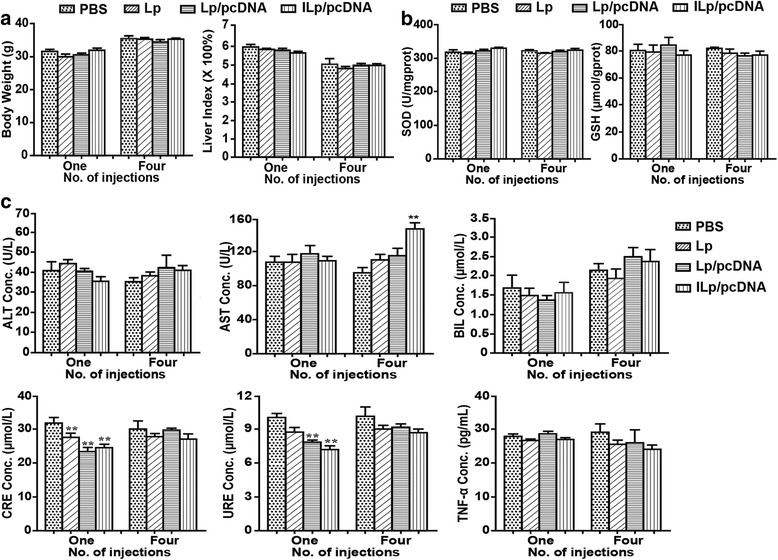
In vivo toxicity investigation. **a** Body weight changes, Liver index (liver weight/body weight, g/g). **b** Glutathione (GSH) and Superoxide dismutase (SOD) assay. **c** Biochemical indices (Alanine aminotransferase [ALT], Aspartate aminotransferase [AST], Total bilirubin [BIL], Creatinine [CRE], and Ureal nitrogen [URE]), and TNF-α levels. The four groups for (**a**, **b**, **c**) were as follows: PBS, Lp, Lp/pcDNA, and ILp/pcDNA group with POPC concentration of 10 mg/kg. Every four days, for a total of four doses with 200 μL of solution for each mouse, the mice were injected in the tail vein. The mice were sacrificed at 5 (one injection) and 17 (four injections) days post administration. Data are presented as the mean ± SD (*n* = 6); ^*^P < 0.05 is considered statistically significant

No differences were observed in mice behavior among any groups after injection, including vocalization, breathing, moving, hunching, or interaction with cage mates. As shown in Fig. 3a, no significant difference was detected in body weight and liver index between groups (*P* > 0.05). Fig. 3b shows the GSH levels and SOD activity in mouse liver at 5 and 17 days after injection of Lp/pcDNA or ILp/pcDNA. The results indicated that there were no significant differences among the four groups.

Concentrations of alanine aminotransferase (ALT), aspartate aminotransferase (AST), total bilirubin (BIL), creatinine (CRE), and ureal nitrogen (URE) in mouse blood at 5 and 17 days after exposure were shown in Fig. 3c. Throughout the entire treatment period, ALT and BIL concentrations in the Lp groups were not significantly different from those of PBS group. AST concentration gradually increased among the Lp groups, and was higher in the IL/pcDNA group than in the PBS group at 17 days after treatment (*P* < 0.05). CRE and URE concentrations significantly decreased (*P* < 0.05) in Lp groups compared with the concentrations reported for the PBS group at 5 days after exposure, indicative of no damage to the kidney. TNF-α is one of the most important promoters of inflammation, necrosis, and fibrosis in liver damage [22], but no significant difference in its expression was observed among the four groups.

The histological changes in the mouse heart, liver, spleen, kidney, and lung in all groups after treatment were studied (data not shown here), and no obvious damage was observed in the tissues of Lp groups at 17 days after exposure compared with that reported for the PBS group.

### In vivo tumor-targeted imaging

**Fig. 4:**
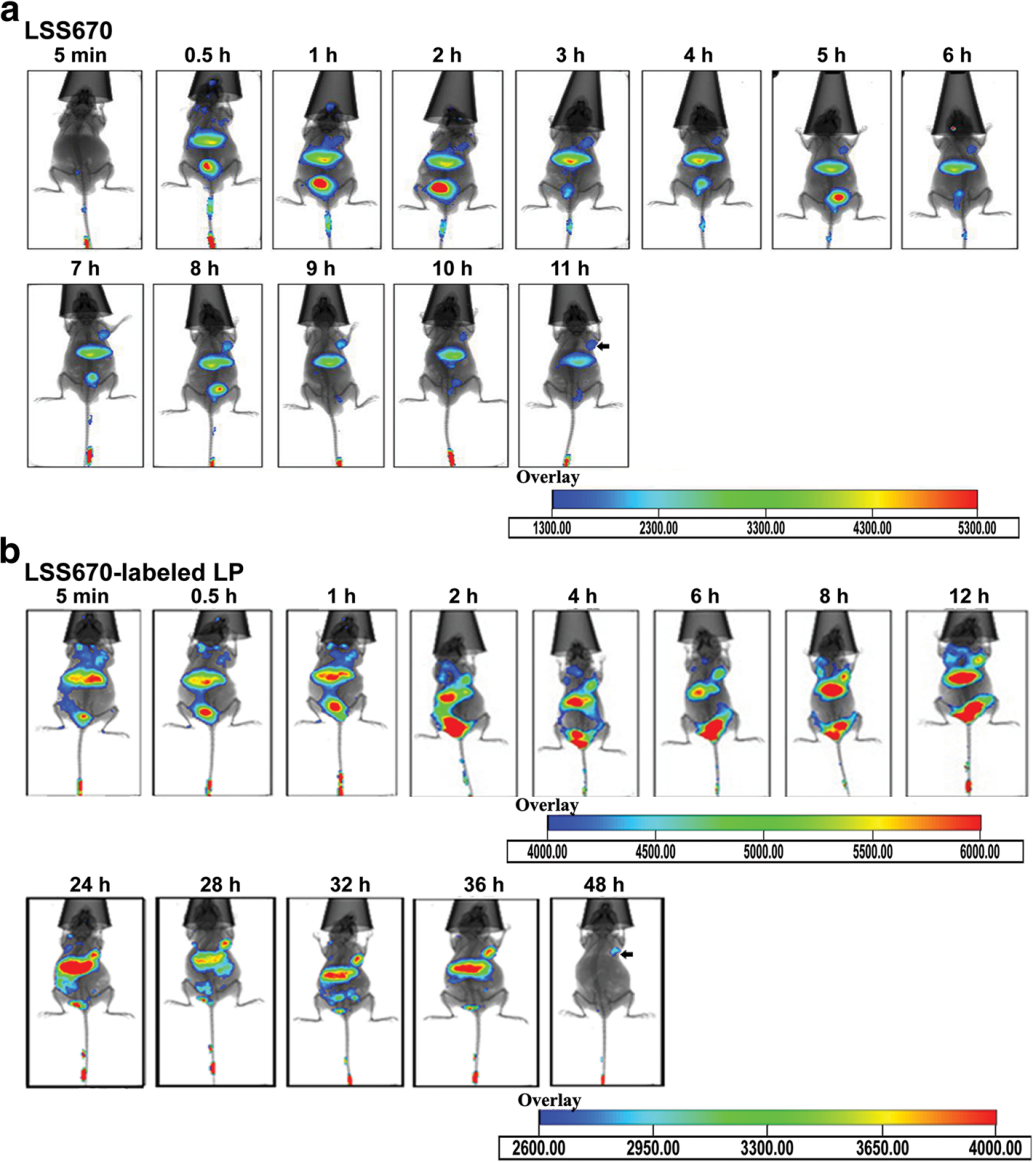
In vivo fluorescent images of nude mice bearing MDA-MB-231-Luc breast cancer xenografts implanted under the right front axilla. **a** After *i.v.* injection of free LSS670 dye. **b** *i.v.* injection of LSS670-labeled Lp. Fluorescent images were acquired from 5 min to 48 h after *i.v.* injection, *n* = 4. Optical imaging was performed with the Kodak in vivo FXPro imaging system, which combines multispectral fluorescence, luminescence, and digital X-ray capabilities in a single system. The excitation and emission filters were set at 650 and 700 nm, respectively

**Fig. 5:**
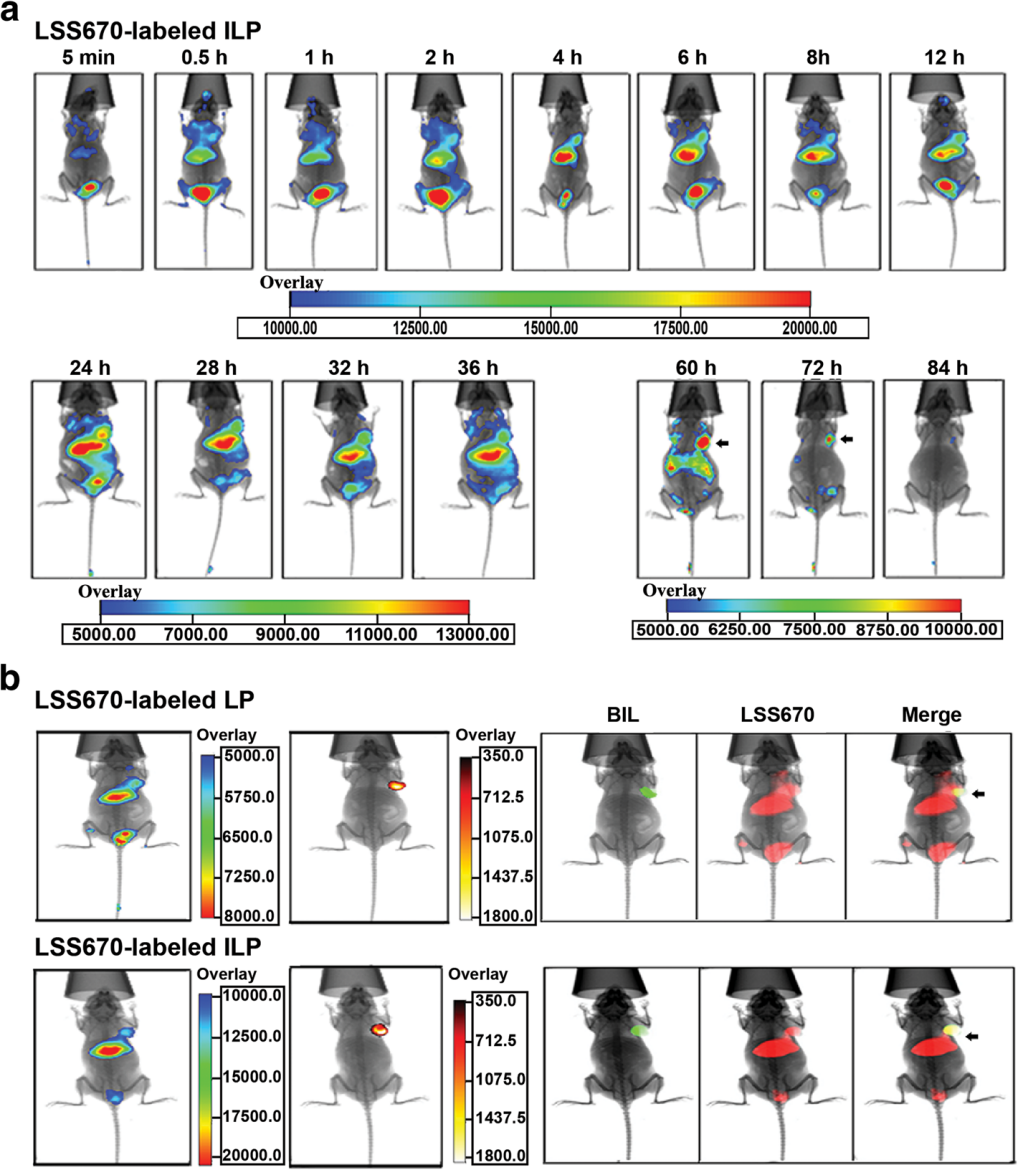
In vivo fluorescent images of nude mice bearing MDA-MB-231-Luc breast cancer xenografts implanted under the right front axilla. **a** After *i.v.* injection of LSS670 labeled ILp, and fluorescence (LSS670, red) and bioluminescence (BLI, green) of nude mice at 12 h after injection of LSS670-labeled Lp or ILp. **b** For bioluminescent imaging, the light-sensitive substrate D-luciferin was administered by intraperitoneal injection of approximately 2.5 mg luciferin/kg body weight for each mouse, and images were captured 10 min after injection. The exposure time was 3 min, with 4 × 4 binning. The black arrow indicates the tumor location

**Fig. 6:**
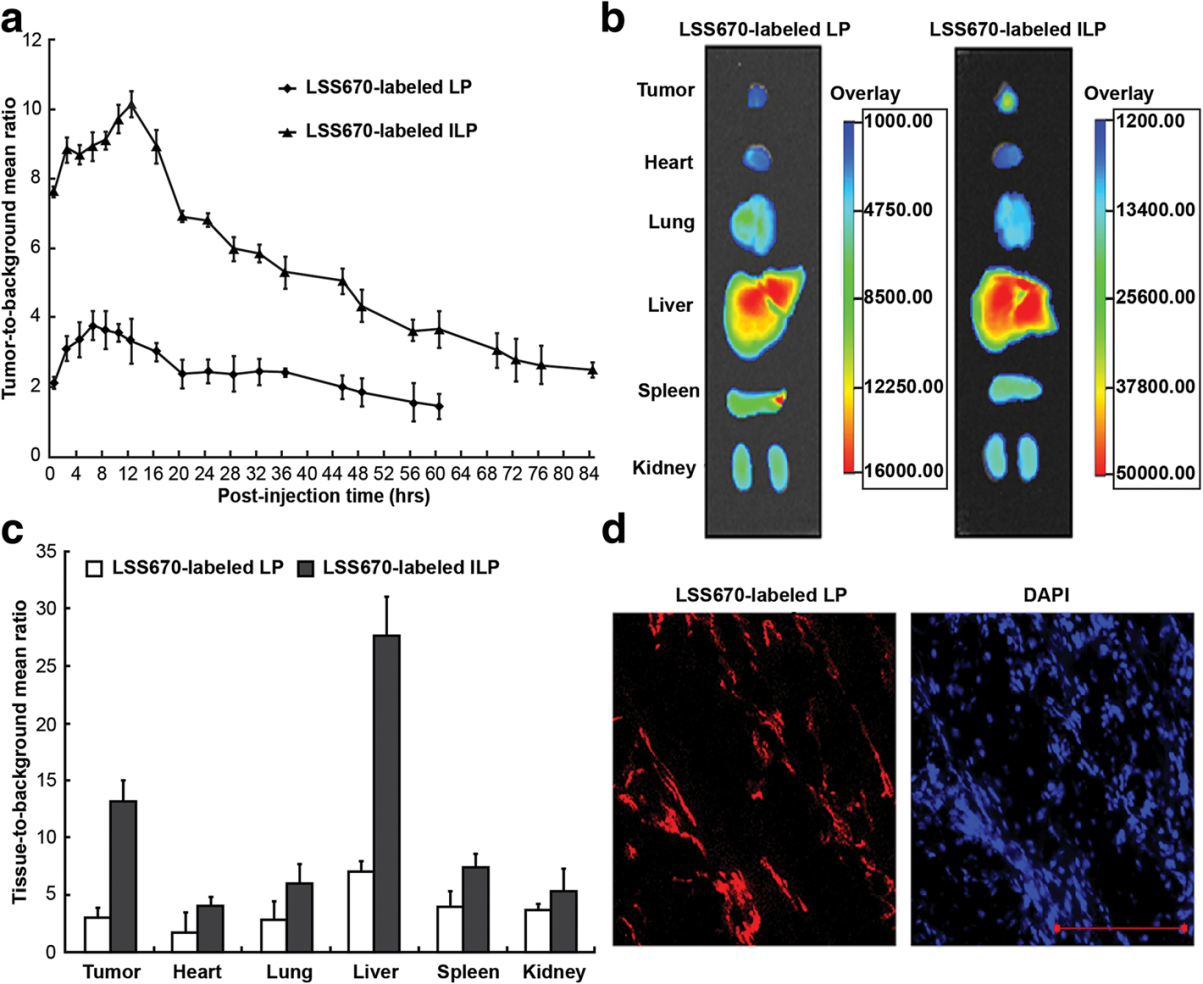
In vivo fluorescence distribution in the mouse xenograft model. **a** Normalized fluorescence of LSS670-labeled Lp and ILp in the mouse xenograft model (*n* = 4) as a function of time. Normalized fluorescence signals (i.e., tumor-to-background signal ratios) were calculated by dividing the total fluorescence intensity in the tumor by the background intensity. **b** & **c** Biodistribution data at 12 h post injection of LSS670 labeled-Lp and ILp in nude mice bearing MDA-MB-231-Luc breast cancer xenografts. **d** Two-color confocal microscopy images of 5-μm sections of frozen tumor tissues harvested 12 h after injection of LSS670-labeled ILp. DAPI, blue); LSS670-labeled ILp, red). Scale bar: 100 μm

To visualize the tumor targeting of ILp, free LSS670 dye, LSS670-labeled Lp, and LSS670-labeled ILp were injected *i.v.* into nude mice bearing MDA-MB-231-Luc xenografts implanted under the right front axilla. As shown in Fig. 4, Lp injection in athymic mice significantly prolonged the circulation of the dye compared with free LSS670, which showed extremely rapid clearance. Almost all free dye was cleared by 11 h after injection. Lp showed more prominent fluorescence than free LSS670 did, with slight accumulation in the tumor (right front axilla, lasting approximately 40 h); however, compared with that for nontargeting nanoparticles, the accumulation of anti-CD105 nanoparticles in the tumor lasted notably longer (up to 72 h after injection), and the intensity of fluorescence was also much stronger (Fig. 5a, see the overlay). The normalized fluorescence intensity (tumor-to-background signal ratio) was estimated from the regions of interest in the tumor and normal tissues at different times after injection (Fig. 6a). At 2 h after injection, fluorescence signals were significant at the tumor site and in mononuclear phagocytic system (MPS) organs, which mediate Lp clearance. In the LSS670-labeled ILp group, tumor fluorescence gradually increased over time up to 12 h and then persisted for more than 72 h, whereas the fluorescent signal decreased to near-baseline levels in the MPS organs. Tumor-specific fluorescent imaging was conducted at 60 h, and continued for at least a further 12 h.

At 12 h after injection, tumor accumulation in the ILp group was at its peak, based on long-term observation results. Tumor-targeting activity was confirmed by bioluminescent imaging of the tumor sites 10 min after luciferin injection. Bioluminescence was readily detected at the tumor sites in all groups, but the bioluminescence images corresponded exactly to the fluorescence images at the tumor sites of the ILp group only (Fig. 5b). Figure 6b,c show the biodistribution data of the Lp at 12 h post injection. Overall, the liver and spleen had a significant biodistribution of Lp (tissue-to-background ratio of 7.1 ± 0.9 and 4.7 ± 1.5, respectively) and ILp (tissue-to-background ratio of 27.5 ± 3.3 and 10.5 ± 1.1, respectively). The biodistribution of ILp in the tumor was higher (tumor-to-background ratio of 13.4 ± 1.7) than that for Lp (tumor-to-background ratio of 2.6 ± 0.8), thus providing good tumor contrast. The difference in biodistribution of ILp and Lp reached statistical significance and confirmed the CD105 specificity of ILp. The tumor xenografts were sectioned, and fluorescence was observed using confocal microscopy. LSS670-labeled ILp showed accumulation and targeting of the microvascular structure in the tumor tissue (Fig. 6d).

### Treatment of mice with pcDNA3.1-CSF1-mES-loaded ILp

**Fig. 7:**
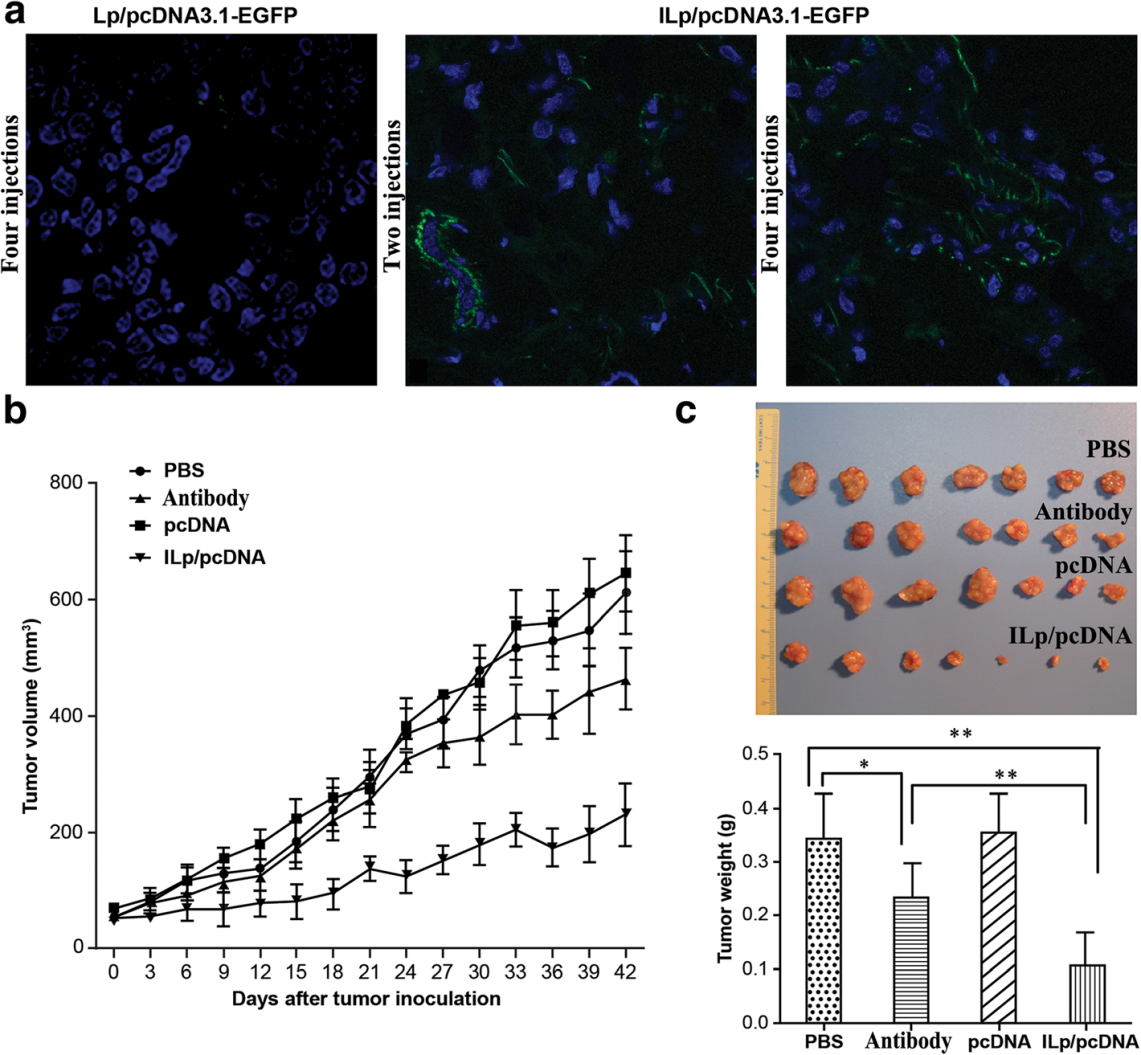
In vivo targeted therapy with pcDNA-loaded ILp. **a** In vivo targeted gene transfection with pcDNA3.1-EGFP-loaded Lp and ILp. Confocal imaging was obtained for Lp after four injection doses at day 14, and for ILp after two doses at day 6 or four doses at day 14 (DAPI/nuclei, blue; EGFP, green). **b** ILp-entrapped pcDNA3.1-CSF1-mES can mediate effective tumor-targeted therapy in vivo. To establish subcutaneous (*s.c.*) tumors, 5 × 10^6^ MDA-MB-231 cells in 0.2 mL of PBS were injected into the mammary fat pad of NOD/SCID mice. The mice were randomly allocated to four groups (*n* = 7), which were intravenously (*i.v.*) treated with 150 μL of either PBS, pcDNA (naked pcDNA3.1-CSF1-mES), antibody (105 μg of anti-CD105 mAb, an amount equal to that in ILp), or ILp/pcDNA. The quantity of pcDNA3.1-CSF1-mES corresponded to 50 μg DNA diluted with PBS solution. The animals were treated four times on days 1, 5, 9, and 13 after tumor establishment. Tumor size was measured by Vernier calipers at 3-day intervals after inoculation with tumor cells. The tumor volume was calculated according to the formula d1 × (d2)^2^ × 0.5, where d1 is the largest diameter and d2 is the perpendicular diameter. The mice were monitored daily for survival. **c** Ex vivo tumor photos and weights. After the last measurement, tumors from the four groups were stripped, imaged, and weighted

MDA-MB-231 tumor-bearing mice were treated with pcDNA3.1-EGFP-loaded Lp or ILp to evaluate the ability of ILp for in vivo transfection, and the confocal imaging results were shown in Fig. 7a. Tumor tissues were stripped at 14 days after four injection doses and almost no green fluorescence was detected in the Lp/pcDNA3.1-EGFP group, although the signal was clearly detected in the ILp/pcDNA3.1-EGFP groups, even at 6 days after two injection doses. The EGFP green fluorescence accumulation pattern observed was similar to that of LSS670-labeled ILp mentioned above. The experimental treatment protocol was therefore deemed suitable for further targeted gene treatment.

Results of in vivo gene therapy were shown in Fig. 7b&c. The average tumor volume of each group at 42 days after injection was 611.85 ± 71.96, 644.69 ± 64.91, 463.33 ± 52.20, and 229.75 ± 53.91 mm^3^, with average tumor weight of 0.336 ± 0.084, 0.354 ± 0.071, 0.233 ± 0.063, and 0.103 ± 0.043 g for the PBS, pcDNA3.1-CSF1-mES, anti-CD105 mAb, and ILp/pcDNA3.1-CSF1-mES groups, respectively. The tumor volumes and tumor weight in group PBS and naked plasmid showed negligible difference, suggesting that free plasmid treatment did not affect the tumor growth. In contrast, the treatment of mice with ILp/pcDNA3.1-CSF1-mES reduced tumor growth more effectively than treatment with PBS, naked plasmid or anti-CD105 mAb (all *P* < 0.05). It is worth mentioning that treatment with anti-CD105 mAb also suppressed tumor growth in mice (*P* < 0.05) in comparison with PBS group. Moreover, the ex vivo tumor weights and tumor photos for the four groups after treatment for 42 days was shown in Fig. 7c. The relative tumor weight ratio in the antibody group versus the PBS control was 68%. The relative tumor weight ratio in the ILp/pcDNA group versus the PBS control or antibody group was 29% and 43%, respectively. The ex vivo results had further verified the significant treatment effect of ILp/pcDNA3.1-CSF1- mES to the tumor in vivo*.*

## Discussion

CD105 expression, contrary to that of CD31, is the highest in rapidly proliferating ECs and is not readily detectable in resting ECs or normal organs [7]. Furthermore, targeted CD105 therapy has minimal delivery barriers, and the destruction of only a small number of tumor ECs can potentially lead to the death of many tumor cells [23]. Overall, CD105 is an attractive target for molecular imaging and cancer therapy.

Recently, CD105-targeted molecular imaging of tumor angiogenesis in vivo has gained considerable interest. Tumor microvessel density (MVD) assessed by CD105 training is the gold standard for clinical evaluation of tumor angiogenesis. Hong et al. have described PET imaging of CD105 expression during tumor angiogenesis with ^64^Cu- [24, 25], ^66^Ga- [26], or multimodal imaging with ^89^Zr and IRDye 800CW dual-labeled CD105 mAb [10]. However, CD105-targeted agents combining cancer drug/gene delivery and imaging in a single nanomaterial have not been widely reported to date.

This study describes an efficient targeted CD105 gene-delivery system that combines imaging and therapeutic modalities in a single nanoparticle. Using fluorescent ILp targeted to tumor ECs offers a noninvasive method for monitoring and quantifying the vascular effects of antitumor angiogenic therapy. A particle size of approximately 120 nm will reduce uptake by the reticuloendothelial system and improve the biodistribution and circulation time of nanomaterials [27]. It has been shown that an almost neutral zeta potential of ILp allows recognition by macrophages to be more easily avoided [26]. We showed that the incorporation of CD105 mAb led to increased binding and internalization of Lp to TECs and significantly improved gene expression compared with that observed with untargeted Lp and free plasmid DNA. The in vitro findings indicate that ILp had superior gene transfection ability for primary TECs, which could prove useful for in vivo treatment.

The safety of nanoparticles is a primary concern and must be established as a prerequisite to in vivo therapy studies. SOD is an important antioxidant enzyme that regulates ROS levels. Tripeptide GSH is an important antioxidant. A change in GSH level is highly sensitive in the assessment of toxicological responses and oxidative stress. ALT, AST, BIL, CRE, and URE are key biochemical parameters of hepatic and kidney injury [28]. TNF-α plays an important role in the activation of the NF-κB pathway as a multifunctional proinflammatory cytokine [29]. There were no significant differences in body weight, liver index, oxidative stress, liver and kidney function, and morphology with Lp treatment, compared with that reported for the PBS group. Lp with low toxicity has the advantage of a phospholipid component and PEG surface. PEG has been widely used to overcome issues of aqueous solubility, thermal and light sensitivity, and minimal systemic bioavailability [30, 31]. Our findings provide basic information on the biocompatibility of PEGylated anti-CD105 ILp, which will be beneficial for further application studies.

As shown by the in vivo imaging results, the nanomaterials were mostly cleared from mice through the hepatobiliary pathway, which showed a significant uptake of LSS670 labeled-Lp and anti-CD105 ILp after intravenous injection. Besides the liver, the spleen and kidney also produced a significant fluorescence signal. Both Lp and anti-CD105 ILp accumulated within tumors, consistent with a previous report [32]. Importantly, anti-CD105 ILp was rapidly accumulated and clearly visible in tumor at 0.5 h post injection. The signal remained stable until 84 h, and was detectable at 60–72 h in tumor for anti-CD105 ILp while background fluorescence decreased to near-baseline levels. The uptake of anti-CD105 ILp was higher than that of Lp. The improved targeting effect translated to a more positive treatment effect. Additionally, prolonged blood circulation may contribute to increased tumor accumulation [33].

The endostatin gene was selected in this study because of its established antiangiogenic properties. Endostatin has been shown to inhibit the proliferation and angiogenesis of ECs through multiple pathways, including the VEGF pathway and downregulation of the expression of anti-apoptotic proteins [34–36]. Therefore, endostatin gene therapy may represent an efficient method for overcoming the issue of protein instability [37]. Secreted endostatin as pcDNA3.1-CSF-mES was used to enhance its function in tumor tissues. In vivo therapy demonstrated that ILp/pcDNA3.1-CSF1-mES could effectively target and accumulate at the tumor site and slow tumor growth up to 70%. It is worth mentioning that treatment with anti-CD105 mAb also suppressed the growth of tumors in mice by 31% on day 42 (*P* < 0.05) of treatment. Similar results have been reported previously. Tsujie et al. found that anti-CD105 mAb SN6j showed significant growth suppression in mice with established tumors of colon-26 and 4 T1 cells in BALB/c mice [38]. The safety, pharmacokinetics, and antitumor activity of TRC105 (anti-CD105 IgG1mAb) alone or in combination with bevacizumab have been assessed in patients with advanced solid tumors [39, 40]. Therefore, the antitumor activity of nanoparticles developed in this study combined the targeted anti-angiogenesis function of endostatin and anti-CD105 mAb.

## Conclusions

A targeted PEGylated Lp conjugated with anti-CD105 mAb and loaded with pcDNA3.1-CSF1-mES was developed for use in targeted vascular endothelial cell gene therapy and tumor imaging. The characterization, cellular association, biocompatibility, tumor-targeting imaging, and gene transfer of Lp and ILp were carefully evaluated in vitro and in vivo. The addition of CD105 mAb to Lp did not significantly affect Lp size and structure, but exhibited an excellent ability to target tumor-derived ECs both in vitro and in vivo. The therapeutic effect of ILp involved combining the targeted anti-angiogenesis function of secreted endostatin and anti-CD105 mAb. Future work should focus on investigating the accuracy and sensitivity of in vivo tumor imaging, image-guided gene delivery, and therapeutic applications. Overall, these data demonstrate the advantages associated with using anti-CD105 mAb-conjugated Lp to enhance tumor imaging and gene transfer, and may be beneficial in the development of efficacious and safe vascular targeting agents for cancer therapy.
