# Correction: Efficient targeted tumor imaging and secreted endostatin gene delivery by antiCD105 immunoliposomes

**DOI:** 10.1186/s13046-026-03846-9

**Published:** 2026-10-09

**Authors:** Huiqin Zhuo, Baoshi Zheng, Jianming Liu, Yong Huang, Huiling Wang, Duo Zheng, Naiquan Mao, Jinyu Meng, Sufang Zhou, Liping Zhong, Yongxiang Zhao

**Affiliations:** 1 Department of Gastrointestinal Surgery, Institute of Gastrointestinal Oncology, Zhongshan Hospital, Xiamen University, Xiamen, Fujian 361004 China; 2 National Center for International Research of Biological Targeting Diagnosis and Therapy/Guangxi Key Laboratory of Biological Targeting Diagnosis and Therapy Research/Collaborative Innovation Center for Targeting Tumor Diagnosis and Therapy, Guangxi Medical University, Nanning, Guangxi 530021 China; 3 Department of Cardiothoracic Surgery, First Affiliated Hospital, Guangxi Medical University, Nanning, Guangxi 530021 China; 4 The Third Xiangya Hospital, Central South University, Changsha, 410083 China; 5 Department of Basic Medicine, Shenzhen Key Laboratory of Translational Medicine of Tumor, School of Medicine, Shenzhen University, Shenzhen, Guangdong 518000 China; 6 Biomedical Polymers Laboratory, Soochow University, Suzhou, Jiangsu 215123 China

**Correction: J Exp Clin Cancer Res 37, 42 (2018)**

**https://doi.org/10.1186/s13046-018-0712-8**

Following publication of the original article [1], the authors identified errors in Fig. 4, specifically, the 8 h group in Fig. 4a and the 48 h group in Fig. 4b which were mistakenly used. Furthermore, the 9 h and 10 h groups in Fig. 4a were mislabeled as the 10 h and 11 h groups and the 11 h group was inadvertently omitted.

The correct figure is presented below:

Incorrect Fig. 4

**Fig. 4:**
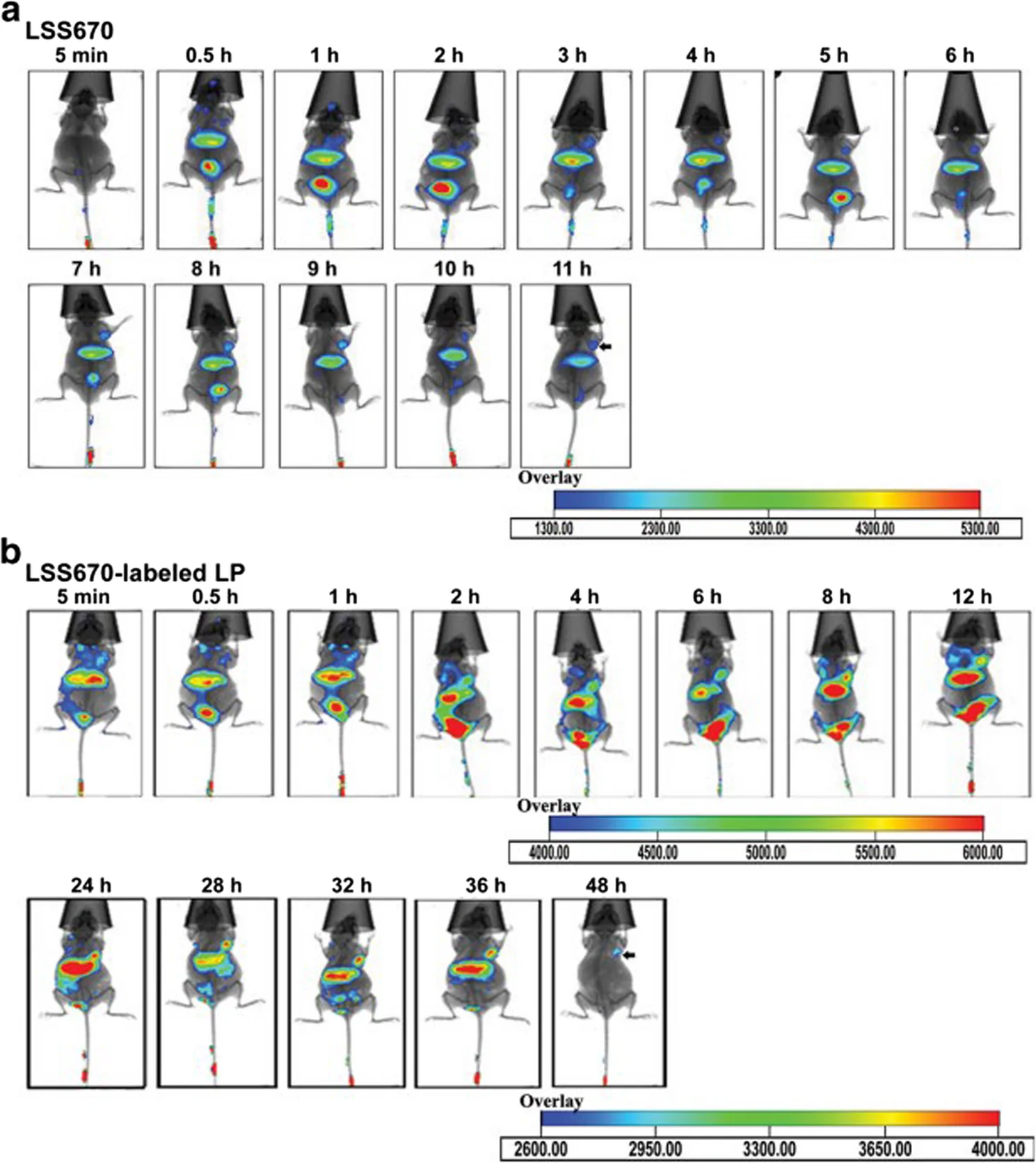
In vivo fluorescent images of nude mice bearing MDA-MB-231-Luc breast cancer xenografts implanted under the right front axilla. **a** After *i.v.* injection of free LSS670 dye. **b***i.v.* injection of LSS670-labeled Lp. Fluorescent images were acquired from 5 min to 48 h after *i.v.* injection, *n* = 4. Optical imaging was performed with the Kodak in vivo FXPro imaging system, which combines multispectral fluorescence, luminescence, and digital X-ray capabilities in a single system. The excitation and emission filters were set at 650 and 700 nm, respectively

Correct Fig. 4

**Fig. 4:**
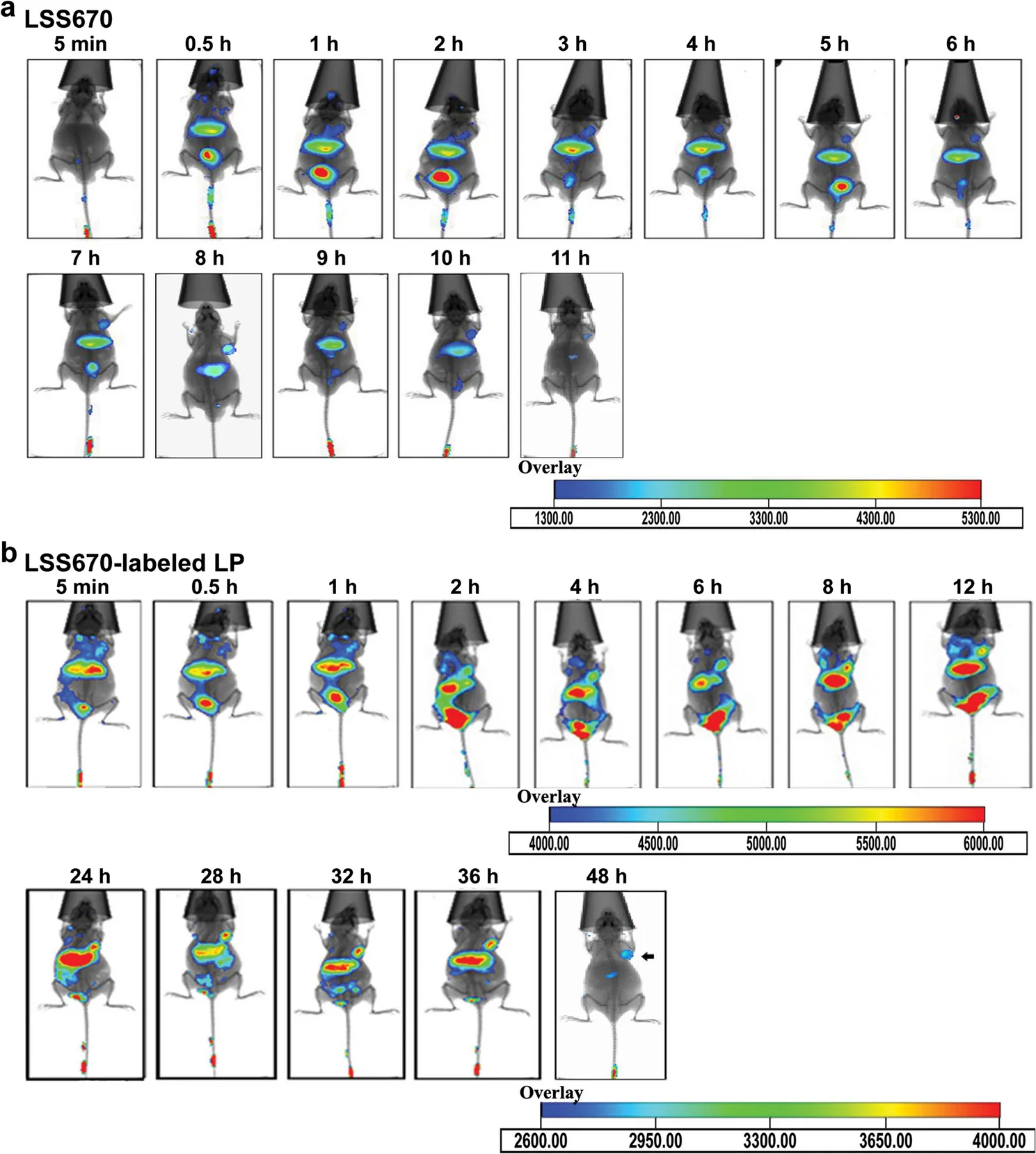
In vivo fluorescent images of nude mice bearing MDA-MB-231-Luc breast cancer xenografts implanted under the right front axilla. **a** After *i.v.* injection of free LSS670 dye. **b***i.v.* injection of LSS670-labeled Lp. Fluorescent images were acquired from 5 min to 48 h after *i.v.* injection, *n* = 4. Optical imaging was performed with the Kodak in vivo FXPro imaging system, which combines multispectral fluorescence, luminescence, and digital X-ray capabilities in a single system. The excitation and emission filters were set at 650 and 700 nm, respectively

The corrections do not compromise the validity of the conclusions and the overall content of the article.
